# Hepatic arterial infusion chemotherapy plus camrelizumab and apatinib for advanced hepatocellular carcinoma

**DOI:** 10.1007/s12072-024-10690-6

**Published:** 2024-07-03

**Authors:** Mengxuan Zuo, Yuzhe Cao, Yi Yang, Guanglei Zheng, Da Li, Hongyan Shao, Qiaoyun Ma, Peng Song, Chao An, Wang Li

**Affiliations:** 1 Department of Minimally Invasive Interventional Therapy, Sun Yat-Sen University Cancer Center, 651 Dongfeng East Road, Guangzhou, 510060 People’s Republic of China; 2 State Key Laboratory of Oncology in South China, Guangzhou, People’s Republic of China; 3 Guangdong Provincial Clinical Research Center for Cancer, Guangzhou, People’s Republic of China; 4 Department of Hepatobiliary Surgery, Cancer Hospital, Chinese Academy of Medical Sciences and Peking Union Medical College, Beijing, People’s Republic of China; 5 Department of Medical Oncology, Chinese PLA General Hospital, Beijing, People’s Republic of China; 6 The Second Medical and National Clinical Research Center for Geriatric Disease, Beijing, People’s Republic of China

**Keywords:** PD-1 inhibitor, VEGFR-2 inhibitor, Combination therapy, Interventional treatment, Overall survival, Progression-free survival, Safety, Propensity score matching, Cox regression analyses, Kaplan–Meier method

## Abstract

**Background and aims:**

There is limited information on combination of hepatic arterial infusion chemotherapy (HAIC) and systemic therapy for advanced hepatocellular carcinoma (Ad-HCC). We aim to compare the efficacy and safety of HAIC plus camrelizumab (a PD-1 inhibitor) and apatinib (an VEGFR-2 inhibitor) versus camrelizumab and apatinib for Ad-HCC.

**Methods:**

From April 2019 to October 2022, 416 patients with Ad-HCC who received either HAIC plus camrelizumab and apatinib (TRIPLET protocol, *n* = 207) or camrelizumab and apatinib (C–A protocol, *n* = 209) were reviewed retrospectively. The propensity score matching (PSM) was used to reduce selective bias. Overall survival (OS) and progression-free survival (PFS) were compared using the Kaplan–Meier method with the log-rank test. Cox regression analyses of independent prognostic factors were evaluated.

**Results:**

After PSM 1:1, 109 patients were assigned to two groups. The median OS of not reached in the TRIPLET group was significantly longer than that of 19.9 months in the C–A group (*p* < 0.001), while in the TRIPLET group, the median PFS of 11.5 months was significantly longer than that of 9.6 months in the C–A group (*p* < 0.001). Multivariate analyses showed that the factors significantly affected the OS were CTP grade, tumor number > 3, and TRIPLET treatment (*p* < 0.001). Grade 3/4 adverse events occurred at a rate of 82.1% vs. 71.3% in TRIPLET and C–A groups, respectively.

**Conclusion:**

The TRIPLET protocol has promising survival benefits in the management of patients with Ad-HCC, with acceptable safety.

*Trail registration*: The study has been retrospectively registered at Chinese Clinical Trial Registry (https://www.chictr.org.cn/, ChiCTR2300075828).

**Supplementary Information:**

The online version contains supplementary material available at 10.1007/s12072-024-10690-6.

## Introduction

As the second most common malignant tumor and the fourth leading cause of cancer death, hepatocellular carcinoma (HCC) has an annual incidence of more than 840,000 new cases globally [1]. Unfortunately, most patients with HCC with liver cirrhosis are already in the advanced stage when they are initially diagnosed, at which point, they have lost the chance to obtain curative therapies [2]. More than 90% of HCC cases in Chinese patients are attributed to hepatitis B virus (HBV) infection, which is thought to be associated with the development of progressive disease and poorer prognoses, with a 5-year overall survival (OS) of only 10–18% [2–4].

Conventional TACE (cTACE) using lipiodol mixed with chemotherapeutics is recommended as the first-line treatment in intermediate-stage HCC [5]. However, a poorer objective response rate (ORR) is commonly found in cases of HCC with a high tumor burden [6]. Hepatic arterial infusion chemotherapy (HAIC) is a promising option that can provide sustained local high concentrations of chemotherapy agents into the tumor and has been shown to outperform the intravenous administration; consequently, HAIC has received extensive support for the effective and safe treatment of intermediate advanced-stage HCC. Zhao Ming et al. have reported that HAIC with the FOLFOX regimen (fluorouracil, leucovorin, and oxaliplatin) displayed an encouraging safety profile and antitumor activity for locally advanced HCC in an open-label, phase III trial [7]. Moreover, for patients with HCC with a mean tumor diameter of 11.2 cm, the median OS was 20.8 months, with an ORR reaching 35.4%. Moreover, in a randomized phase III trial, Shi Ming et al. showed that HAIC with the FOLFOX regimen yielded a higher median OS (23.1 months) and ORR (48%) than cTACE treatment in large HCC (largest diameter > 7 cm) in BCLC A or B stage [8].

In recent years, the efficacy of combining immune checkpoint inhibitors (ICIs) and multitargeted tyrosine kinase inhibitors (TKIs) has attracted increasing interest as an anti-cancer therapy [9–12]. Several active intrinsic immune evasion pathways, including overexpression of vascular endothelial growth factor (VEGF), have been linked to the development and progression of HCC. Anti­VEGF agents reduce VEGF­mediated immunosuppression and induce blood vessel normalization within the tumor and its microenvironment, which may enhance anti-PD-1 efficacy by reversing VEGF­mediated immunosuppression and promoting T­cell infiltration in tumors [13]. An open-label, multicenter, phase 2 trial (RESCUE) using camrelizumab (a PD-1 inhibitor) along with apatinib (an VEGFR-2 inhibitor) as first-line therapy for treating patients with Ad-HCC presented at ESMO 2020 reported a median OS of 20.3 months (95% CI 15.0–NR) with an overall response rate of 34% per RECIST 1.1 and 46% per mRECIST [14]. Moreover, another open-label, multicenter, phase 3 trial (CARES-310) reported that camrelizumab plus ribonucleotide (apatinib) showed a statistically significant and clinically meaningful survival benefit compared with sorafenib for patients with unresectable HCC [15]. Based on the CARES-310 study, China National Medical Products Administration (NMPA) has approved apatinib plus camrelizumab as the first-line treatment of Ad-HCC. Different from Europe and America, the local hepatic treatments including transcatheter arterial chemoembolization (TACE) and radiotherapy were recommended for the treatment of Ad-HCC by China Liver Cancer Staging (CNLC) guideline due to the relatively heavy hepatic tumor burden. A prospective, phase II, open-label study on the TRIPLET protocol has demonstrated the ideal efficacy and relatively acceptable safety of the treatment (ORR per RECIST v1.1: 77.1%, mPFS: 10.38 months, no grade 5 AEs) [16].

Considering the success of these combined therapies, we designed a combination protocol by combining HAIC of FOLFOX with camrelizumab plus apatinib (TRIPLET protocol) for advanced HCC. In this study, we investigated the effectiveness and safety of the TRIPLET protocol and camrelizumab and apatinib treatment in Ad-HCC.

## Materials and methods

The treatment decision was principally made by the patient and their family under the recommendations of interventional oncologists and surgeons. Informed consent was waived because of the retrospective nature of the study. Starting from the onset of combination treatment, all patients were routinely assessed for safety and treatment response. All patients were followed up for the evaluation of toxicity and treatment outcome under the approval of the hospital ethics committee of SYSUCC (B2023-411). This study was conducted in accordance with the principles of the Declaration of Helsinki.

### Patients’ inclusion

This retrospective study reviewed 922 consecutive patients with Ad-HCC who received combined therapy at three hospitals from April 1, 2019, to October 31, 2022. The inclusion criteria were as follows: (1) patients were aged 18–75 years; (2) the patients with HCC were clinically or pathologically confirmed to have BCLC Stage B or C HCC; (3) Eastern Cooperative Oncology Group performance status of 0 or 1; (4) adequate hepatic function with Child–Pugh A or B7; (5) at least one intrahepatic evaluable tumor; and (6) the patients received HAIC plus camrelizumab and apatinib (TRIPLET group) or camrelizumab and apatinib (C–A group). The exclusion criteria were as follows: (1) active or prior autoimmune disease; history of immunosuppressive agent use; (2) history of any other PD-L1/PD-1 antagonist treatment; (3) history of any local treatments including liver section, tumor ablation or transhepatic arterial chemoembolization; (4) HCC combined with other malignancies; (5) simultaneous treatment of TACE combined with HAIC; and (6) incomplete clinical and follow-up data.

The patients in the C–A group all met the following requirements: (1) at least one time camrelizumab injection; (2) at least 1 month apatinib treatment; (3) the injection of camrelizumab occurred the administration of apatinib; (4) during the apatinib plus camrelizumab treatment without any local treatments including ablation, TACE and HAIC. By contrast, the patients included in the TRIPLET group met the following criteria: (1) at least one time camrelizumab injection; (2) at least 1 month apatinib treatment; (3) at least 1 HAIC circle; (4) the injection of camrelizumab occurred during the administration of apatinib; (5) HAIC was performed during or in the previous week to the administration of apatinib; (6) during the apatinib plus camrelizumab treatment without any local treatments including ablation and TACE. All participating institutions strictly followed the inclusion and exclusion criteria to ensure consistency in the baseline characteristics of the population. The treatment regimen for patients would be recommended by the multi-disciplinary team (MDT) that consist of surgical oncologists, radiotherapist, diagnostic radiologist and interventional radiologist. Final decision-making was dominated together with patients and their family members according to patients’ willingness and economic condition.

### Treatment regimen

For the HAIC procedure, a 5-French Yashiro or right hepatic catheter was inserted through the femoral artery with a 2.7-French microcatheter inside. The tip of the microcatheter was located in the tumor feeding artery on day 1 in every treatment cycle. The location of the tip was dependent on the arterial supply of the tumor identified by arteriography: the right/left hepatic artery for tumors in the right/left lobe, and the proper hepatic artery for tumors in two lobes. When the tumor accepted blood supply from extrahepatic arteries simultaneously, such as the celiac trunk or the superior mesenteric artery, the tip was located in the superior feeding artery and the sub-superior arteries were embolized. If necessary, the gastroduodenal artery was embolized using coil embolization. The administration of medication was initiated within 3 days of catheter insertion. The therapeutic scheme was a modified FOLFOX7 regimen, including oxaliplatin (85 mg/m^2^ infusion for 3 h on day 1), leucovorin (400 mg/m^2^ for 2 h from 4 to 5 h on day 1), and fluorouracil (2,500 mg/m^2^ continuous 46-h infusion on days 1 to 3). All chemotherapeutic agents were delivered via HAIC. The catheter and sheath were removed after the completion of HAIC and reinserted for the next HAIC cycle. The criteria for protocol discontinuation are described in Supplementary Information E1.1.

Apatinib (Jiangsu Hengrui Medicine Co. Ltd), a selective VEGFR-2 inhibitor was continuously administered orally at the dose of 250 mg once a day. Camrelizumab, (Jiangsu Hengrui Medicine Co. Ltd), a programmed cell death protein-1 (PD-1) blocker was administered intravenously at the dose of 200 mg each 21-day. The apatinib and camrelizumab treatment would be interrupted when the occurrence of progressive disease or unacceptable toxicity. Dose reduction for apatinib and chemotherapy agents was allowed because of unacceptable toxicity. Meanwhile, if the patients were diagnosed with hepatitis B virus (HBV) infection, he or she would receive antiviral therapy: the oral administration of entecavir or tenofovir. In addition, serum HBV DNA test would be carried out to adjust the regimen of antiviral therapy.

### Data collection and follow-up protocol

To maintain the balance between the TRIPLET and C–A groups and analyze the risk factors associated with survival outcomes, we collected 29 clinical variables related to the enrolled patients. The variable definitions are described in Supplementary Information E1.2. In this study, enrolled patients were censored at the last follow-up date (June 30, 2023). After a thorough TRIPLET procedure was accomplished, the serum alpha-fetoprotein (AFP) and dynamic contrast-enhanced images were examined again at 1–3-month intervals during TRIPLET and at approximately 3-month intervals in the first year and every 6-month intervals thereafter. If suspected metastasis was encountered, chest computed tomography (CT), whole-body bone scans, or positron emission tomography (PET)-CT were performed selectively. The post-study treatment in follow-up is shown in Table S1.

### Outcomes and safety

The responses to combined therapy were assessed by dynamic contrast-enhanced CT or magnetic resonance imaging (MRI) by the investigator as per the modified RECIST (mRECIST), including complete response (CR), partial response (PR), stable disease (SD), and progression disease (PD), which was evaluated independently by two radiologists with 10 years of experience who were blinded to the combined therapy procedures at the time of data collection. In this study, the outcome measures were overall survival (OS) and progression-free survival (PFS). OS was calculated from the date of initial treatment to the date of death from any cause or the deadline for follow-up. The date from the first treatment to the date of PD or the end of follow-up was applied for the calculation of PFS. The other outcomes we assessed were the ORR and disease control rate (DCR). The ORR was defined as the percentage of patients with CR and PR lasting more than 4 weeks from the first radiological confirmation, while the DCR was defined as the percentage of patients with CR, PR, and SD.

Safety assessments were based on symptom, laboratory tests and vital signs during therapy and follow-ups. Outpatient and telephone follow-up by special clinical assistants every 3–6 months were finished. Adverse events (AEs) were evaluated on the basis of Common Terminology Criteria for Adverse Events v5.0. Assessments.

### Statistical analysis

Statistical analysis was performed using SPSS version 25.0 (IBM Corp., NY, USA) and the R software version 4.2.2 (http://www.r-project.org/). The quantitative variables with mean ± standard deviation or median with range or interquartile range (IQR) were compared by the Student t-test. The qualitative variables with frequency were compared using the 2 test (or Fisher exact test, if applicable). The factors including vascular invasion, extrahepatic metastasis, CTP grade and BCLC stage which may influence the survival and tumor response were chosen to perform propensity score matching (PSM). We applied 1:1 PSM using a nearest-neighbor algorithm with the 0.03 caliper width to adjust the potential unbalance variables which may influence the patients’ prognosis in both treatment groups. The survival results were compared using the Kaplan–Meier method with log-rank test. Univariate analyses of independent prognostic factors were evaluated by means of the Cox regression model, and then multivariable analyses were applied with the factors showing significance in the univariate analyses.

All tests of significance were two-sided and a *p* < 0.05 was interpreted to carry statistical significance.

## Results

### Patients enrolled

**Fig. 1:**
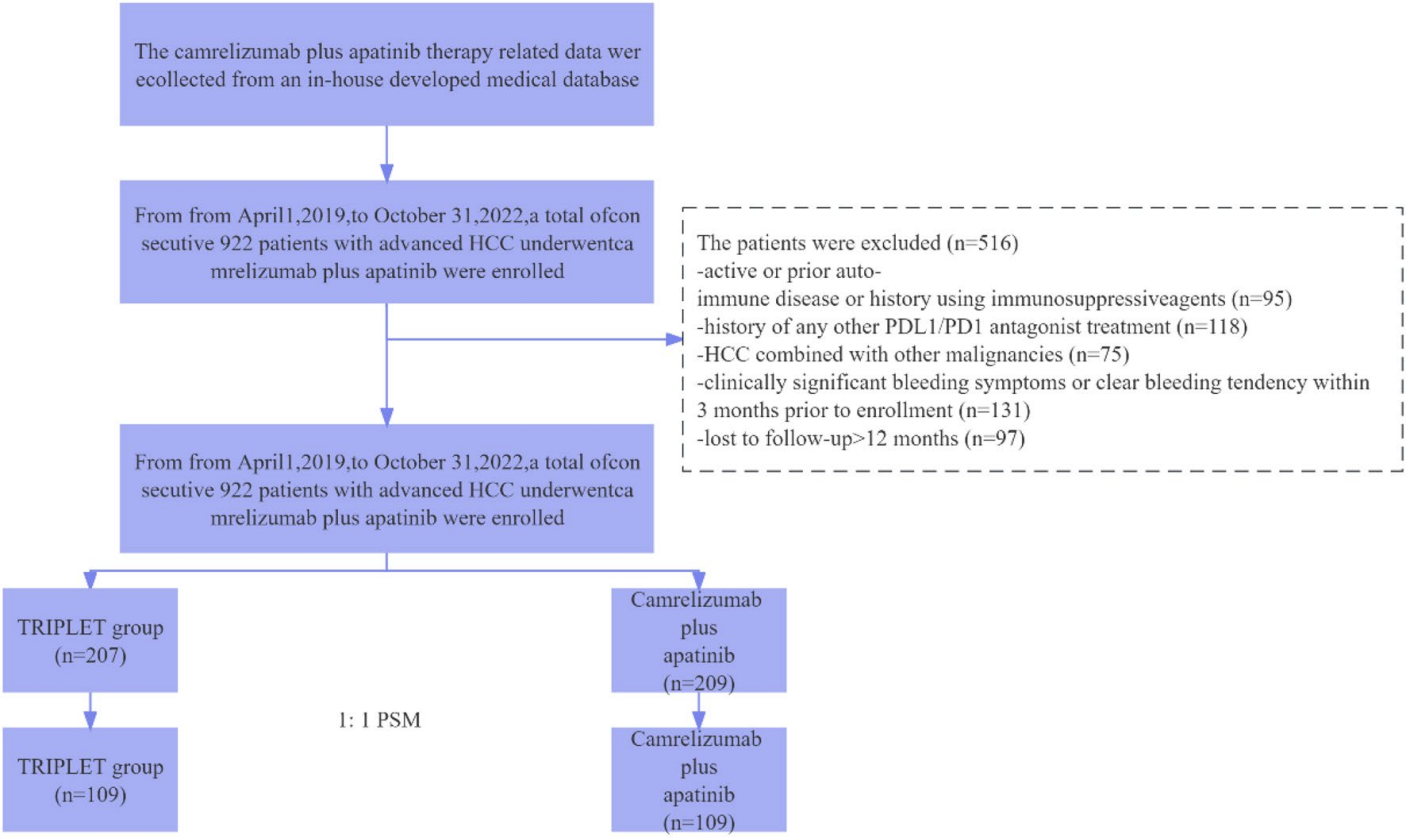
Patients’ selection flow. HCC: hepatocellular carcinoma

Figure 1 demonstrates the enrollment pathways of patients with Ad-HCC who underwent combined therapy. Finally, 416 treatment-naïve patients with HCC (43 females and 373 males; mean age, 50.7 ± 10.1 years) were reviewed in the study. Among them, 207 patients were included in the TRIPLET (T) group, and 209 patients were included in the camrelizumab and apatinib (C–A) groups. The baseline characteristics stratified by combined therapy modality are outlined in Table 1. Overall, 88.2% patients in Barcelona Clinic Liver Cancer (BCLC) C stage, 86.2% had hepatitis B virus etiology, 28.3% had an AFP concentration of > 400 ng/ml. Several clinical variables including Child–Turcotte–Pugh (CTP) grade, tumors’ maximum diameter and number, AFP, vascular invasion, metastasis, BCLC stage, and ALBI grade have significantly different distribution between T group and C–A group (*p* < 0.05). After 1:1 PSM, the variables have no significant statistical difference.

**Table 1:** Baseline characteristics of the patients with Ad-HCC who received TRIPLET or camrelizumab plus apatinib

Variables	Unmatched			PSM (1:1)		
	TRIPLET	Camrelizumab plus apatinib	*p* value	TRIPLET	Camrelizumab plus apatinib	*p* value
	(*n* = 207)	(*n* = 209)		(*n* = 109)	(*n* = 109)	
Age, years						
≤ 65	190 (91.8%)	181 (86.6%)	0.122	101 (92.7%)	93 (85.3%)	0.13
> 65	17 (8.2%)	28 (13.4%)		8 (7.3%)	16 (14.7%)	
Sex						
Female	22 (10.6%)	21 (10.0%)	0.973	16 (14.7%)	12 (11.0%)	0.544
Male	185 (89.4%)	188 (90.0%)		93 (85.3%)	97 (89.0%)	
ECOG-PS						
0–1	207 (100%)	209 (100%)	1	109 (100%)	109(100%)	1
Hepatitis B virus						
Negative	14 (6.8%)	18 (8.6%)	0.600	6 (5.5%)	11 (10.1%)	0.312
Positive	193 (93.2%)	191 (91.4%)		103 (94.5%)	98 (89.9%)	
Liver cirrhosis						
Presence	201	199	0.317	106	104	0.471
Viral	193	191		103	98	
Alcoholic or biliary	8	8		3	6	
Absence	6	10		3	5	
CTP						
A	187 (90.3%)	209 (100%)	< 0.001	109 (100%)	109 (100%)	1
B	20 (9.7%)	0 (0%)		0 (0%)	0 (0%)	
HCC number						
1–3	94 (45.4%)	155 (74.2%)	< 0.001	41 (37.6%)	82 (75.2%)	< 0.001
> 3	113 (54.6%)	54 (25.8%)		68 (62.4%)	27 (24.8%)	
Tumor maximum diameter, cm(mean (SD))	10.3 (4.27)	4.59 (3.27)	< 0.001	10.2 (4.61)	5.32 (3.80)	< 0.001
AFP, ng/ml						
≤ 400	99 (47.8%)	199 (95.2%)	< 0.001	46 (42.2%)	103 (94.5%)	< 0.001
> 400	108 (52.2%)	10 (4.8%)		63 (57.8%)	6 (5.5%)	
Vascular invasion						
Absence	58 (28.0%)	151 (72.2%)	< 0.001	51 (46.8%)	51 (46.8%)	1
Presence	149 (72.0%)	58 (27.8%)		58 (53.2%)	58 (53.2%)	
Extrahepatic metastasis						
Absence	121 (58.5%)	47 (22.5%)	< 0.001	40 (36.7%)	40 (36.7%)	1
Presence	86 (41.5%)	162 (77.5%)		69 (63.3%)	69 (63.3%)	
BCLC stage						
B	15 (7.2%)	34 (16.3%)	0.00689	9 (8.3%)	9 (8.3%)	1
C	192 (92.8%)	175 (83.7%)		100 (91.7%)	100 (91.7%)	
ALB [mean (SD)]	41.0 (4.55)	42.5 (4.33)	< 0.001	41.1 (4.12)	41.9 (4.49)	0.214
ALT [mean (SD)]	59.2 (73.0)	35.3 (19.3)	< 0.001	49.7 (29.1)	35.6 (21.5)	< 0.001
AST [mean (SD)]	86.9 (92.8)	43.4 (32.2)	< 0.001	80.7 (78.3)	47.8 (41.0)	< 0.001
TBIL [mean (SD)]	19.3 (14.8)	15.2 (6.67)	< 0.001	17.3 (8.09)	16.4 (7.76)	0.406
INR [mean (SD)]	1.07 (0.119)	1.04 (0.0852)	0.0306	1.06 (0.109)	1.05 (0.0895)	0.48
PLT [mean (SD)]	248 (120)	166 (60.7)	< 0.001	266 (135)	169 (63.5)	< 0.001
ALBI						
1	118 (57.0%)	148 (70.8%)	0.009	65 (59.6%)	68 (62.4%)	1
2	88 (42.5%)	61 (29.2%)		44 (40.4%)	41 (37.6%)	
3	1 (0.5%)	0 (0%)		0 (0%)	0 (0%)	

Data are number of patients; data in parentheses are percentage unless otherwise indicated. Data in bracket are percent of patients. The quantitative data with mean ± standard deviation were compared by the Student t-test. The qualitative data in two groups were compared using the Chi square test

Ad**-**HCC: advanced hepatocellular carcinoma; TRIPLET: hepatic arterial infusion chemotherapy and camrelizumab plus apatinib; PSM: propensity score match; ECOG-PS Eastern Cooperative Oncology Group Performance Status; CTP: Child–Turcotte–Pugh; AFP: α-fetoprotein; ALBI: albumin-bilirubin grade; ALB: albumin; ALT: alanine aminotransferase; AST: aspartate aminotransferase; PT: prothrombin time; INR: international normalized ratio; TBIL: total bilirubin; PLT: platelet; BCLC: Barcelona Clinic Liver Cancer

*p* < 0.05 suggests statistically significant differences

### Antitumor activity

The antitumor activity comparison between the T and C–A groups is shown in Table 2**.** After PSM 1:1, the optimal ORR in T group was higher significantly than those in C–A group (69.7% vs. 38.5%, *p* < 0.001). 12.1% (25/207) HCC patients occurred downstage after TRIPLET, showing significantly higher than that (4.5%) in C–A group (*p* < 0.001). Moreover, the conversion surgery rate in the T group was significantly higher than that in the C–A group (*p* < 0.001). Notably, curative surgical resection after TRIPLET was conducted in 54 patients (26.2%, 54/207) in the T group and 16 patients (7.7%, 16/209) in the C–A group, owing to intrahepatic tumor shrinkage which made the patients meet the Milan criteria.

**Table 2:** Response comparison of Ad-HCC patient according to treatment modality

Variables	TRIPLET vs C–A (unadjusted)			TRIPLET vs C–A (PSM 1:1)		
	TRIPLET (*n* = 207)	C–A (*n* = 209)	*p* value	TRIPLET (*n* = 109)	C–A (*n* = 109)	*p* value
Tumor response						
ORR	139 (67.1%)	73 (34.9%)	< 0.001	76 (69.7%)	42 (38.5%)	< 0.001
DCR	174 (84.0%)	161 (77.0%)	< 0.001	93 (85.3%)	88 (80.7%)	< 0.001
CR	21 (10.1%)	8 (3.8%)		10 (9.2%)	5 (4.6%)	
PR	118 (57.0%)	65 (31.1%)		66 (60.5%)	37 (33.9%)	
SD	35 (16.9%)	88 (42.1%)		17 (15.6%)	46 (42.2%)	
PD	33 (16.0%)	48 (23.0%)		16 (14.7%)	21 (19.3%)	

Data are number of patients; data in parentheses are percentage unless otherwise indicated and data in bracket are percent of patients

Ad**-**HCC: advanced hepatocellular carcinoma; TRIPLET: hepatic arterial infusion chemotherapy and camrelizumab plus apatinib; C–A: camrelizumab plus apatinib; HR: hazard ratio; CI: confidence interval

*p* < 0.05 suggests statistically significant differences

### Oncological outcome comparison

**Fig. 2:**
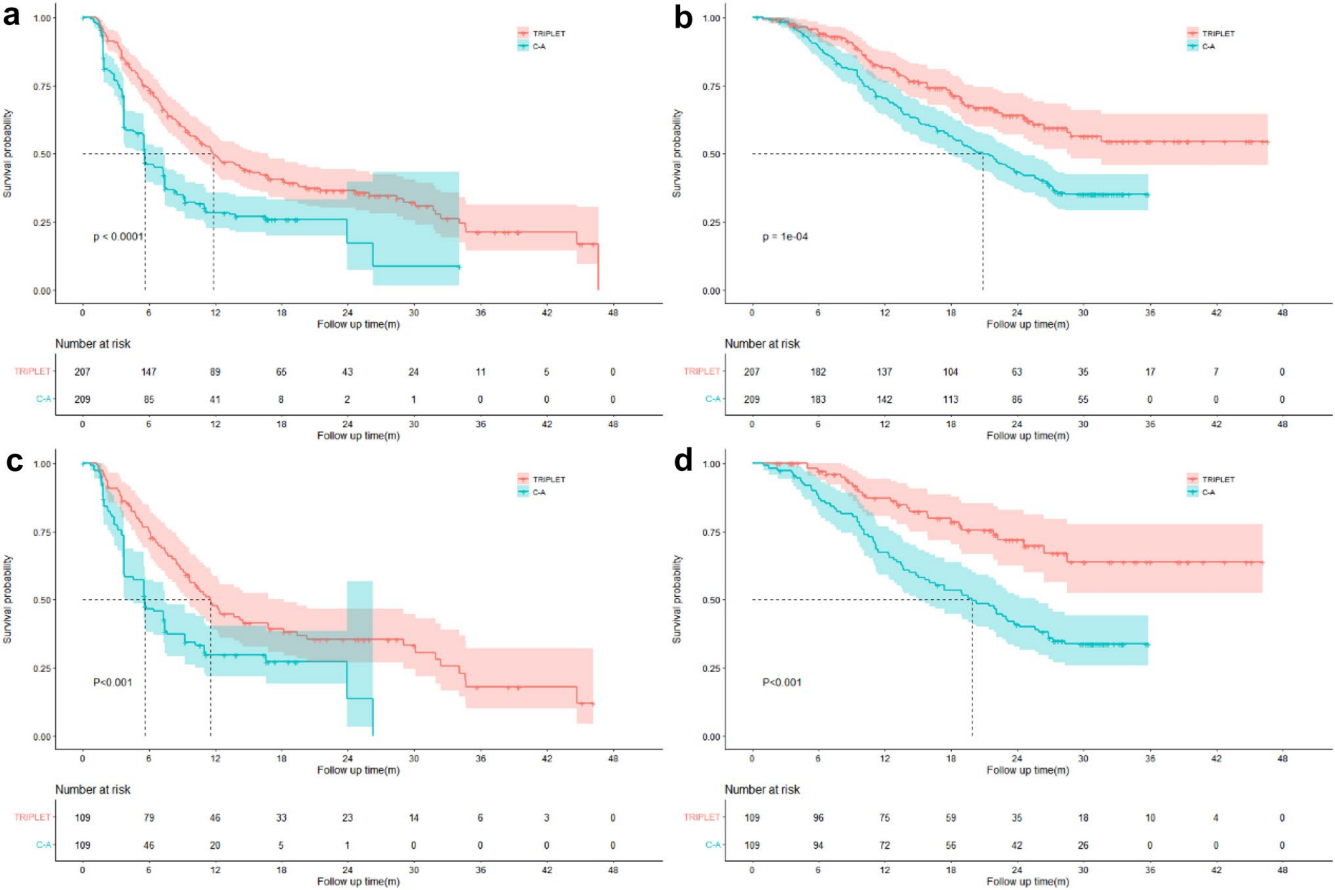
Kaplan–Meier curves of cumulative 2-year overall survival rate and 2-year progression-free survival rate in two different groups. **a** Cumulative 2-year progression-free survival rate before propensity score matching; **b** cumulative 2-year overall survival rate before propensity score matching; **c** cumulative 2-year progression-free survival rate after propensity score matching; **d** cumulative 2-year overall survival rate after propensity score matching

The median follow-up duration for the T and C–A groups was 22.8 months (IQR: 8.2–61.3 months) and 24.5 months (IQR: 6.4–48.8 months), respectively. In the crude Kaplan–Meier analyses, significant differences were observed with regard to PFS (median PFS: T, 11.8 months vs C–A, 5.8 months; *p* < 0.001; Fig. 2A) and OS (median OS: T, Not reached vs C–A, 20.9 months; *p* < 0.001; Fig. 2B). The PSM adjusted Kaplan–Meier analyses also showed that significant differences were observed with regard to PFS (median PFS: T, 11.5 months vs C–A, 5.6 months; *p* < 0.001; Fig. 2C) and OS (median OS: T, Not reached vs C–A, 19.9 months; *p* = 0.001; Fig. 2D).

### Risk factors for survival outcomes

The risk factors for OS and PFS were assessed by univariate and multivariate analyses prior to the PSM (Table 3). In the univariate analyses, CTP-B [hazard ratio (HR): 2.86; 95% confidence interval (CI) 1.67–5.87; *p* < 0.001], tumor number > 3 (HR: 1.39; 95% CI 1.04–1.85; *p* = 0.024), metastasis (HR: 10.44; 95% CI 1.07–1.93;* p* = 0.017), and camrelizumab plus apatinib (HR: 1.79; 95% CI 1.33–2.40; *p* < 0.001) were significant factors for poor OS. Multivariate analyses showed that the factors that significantly affected the OS rate were CTP-B (HR: 4.73; 95% CI 2.50–8.94; *p* < 0.001), tumor number > 3 (HR: 1.72; 95% CI 1.28–2.32; *p* < 0.001), and camrelizumab plus apatinib (HR: 2.34; 95% CI 1.64–3.32; *p* < 0.001). In the univariate analyses, ECOG-PS 1 (HR: 1.53; 95% CI 1.14–2.06; *p* = 0.005), tumor number > 3 (HR: 1.30; 95% CI 1.03–1.66; *p* = 0.029), metastasis (HR: 1.50; 95% CI 1.17–1.91; *p* = 0.001), and camrelizumab plus apatinib (HR: 1.83; 95% CI: 1.43–2.35; *p* < 0.001) were significant factors for poor PFS. Multivariate analyses showed that tumor number > 3 (HR: 1.56; 95% CI 1.22–2.01; *p* < 0.001) and camrelizumab plus apatinib (HR: 1.96; 95% CI 1.44–2.65; *p* < 0.001) significantly affected the PFS rate.

**Table 3:** Prognostic factor analysis for overall survival and progression-free survival prior to propensity scores matching

Variables	OS		PFS	
	Univariable	Multivariable	Univariable	Multivariable
	HR (95% CI, *p* value)	HR (95% CI, *p* value)	HR (95% CI, *p* value)	HR (95% CI, *p* value)
Age, years				
≤ 65	Ref		Ref	
> 65	1.02 (0.65–1.59, *p* = 0.939)		0.71 (0.46–1.09, *p* = 0.114)	
Gender				
Female	Ref		Ref	
Male	1.60 (0.96–2.67, *p* = 0.072)		1.22 (0.82–1.82, *p* = 0.320)	
ECOG-PS				
0	Ref		Ref	Ref
1	1.36 (0.99–1.86, *p* = 0.059)		1.53 (1.14–2.06, *p* = 0.005)	1.02 (0.73–1.42, *p* = 0.911)
HBV				
Absence	Ref		Ref	
Presence	0.99 (0.67–1.48, *p* = 0.978)		0.88 (0.63–1.25, *p* = 0.479)	
CTP				
A	Ref	Ref	Ref	
B	2.80 (1.56–5.05, *p* < 0.001)	4.73 (2.50–8.94, *p* < 0.001)	1.16 (0.65–2.06, *p* = 0.622)	
HCC number				
1–3	Ref	Ref	Ref	Ref
> 3	1.39 (1.04–1.85, *p* = 0.024)	1.72 (1.28–2.32, *p* < 0.001)	1.30 (1.03–1.66, *p* = 0.029)	1.56 (1.22–2.01, *p* < 0.001)
HCC diameter, cm				
≤ 5	Ref		Ref	
> 5	1.07 (0.80–1.41, *p* = 0.656)		0.79 (0.62–1.01, *p* = 0.062)	
AFP, ng/ml				
≤ 400	Ref		Ref	
> 400	0.89 (0.64–1.24, *p* = 0.493)		0.87 (0.68–1.13, *p* = 0.306)	
Vascular invasion				
Absence	Ref		Ref	
Presence	1.08 (0.81–1.43, *p* = 0.601)		0.90 (0.71–1.14, *p* = 0.392)	
Metastasis				
Absence	Ref		Ref	Ref
Presence	1.44 (1.07–1.93, *p* = 0.017)		1.50 (1.17–1.91, *p* = 0.001)	1.16 (0.89–1.52, *p* = 0.265)
BCLC stage				
B	Ref	Ref	Ref	
C	1.03 (0.68–1.56, *p* = 0.881)	1.07 (0.78–1.46, *p* = 0.692)	0.94 (0.65–1.35, *p* = 0.726)	
Treatment protocol				
TRIPLET	Ref	Ref	Ref	Ref
Camrelizumab plus apatinib	1.79 (1.33–2.40, *p* < 0.001)	2.34 (1.64–3.32, *p* < 0.001)	1.83 (1.43–2.35, *p* < 0.001)	1.96 (1.44–2.65, *p* < 0.001)

A Cox proportional hazards regression model for survival was used. All variables were included in a multivariate stepwise Cox regression analysis. Only the variables with a *p* < 0.05 in the final model were presented

HR: hazard ratio; CI: confidence intervals; HBV: hepatitis B virus; AFP: α-fetoprotein; Ad-HCC: advanced hepatocellular carcinoma; TRIPLET: hepatic arterial infusion chemotherapy and camrelizumab plus apatinib

### Subgroup analysis

**Fig. 3:**
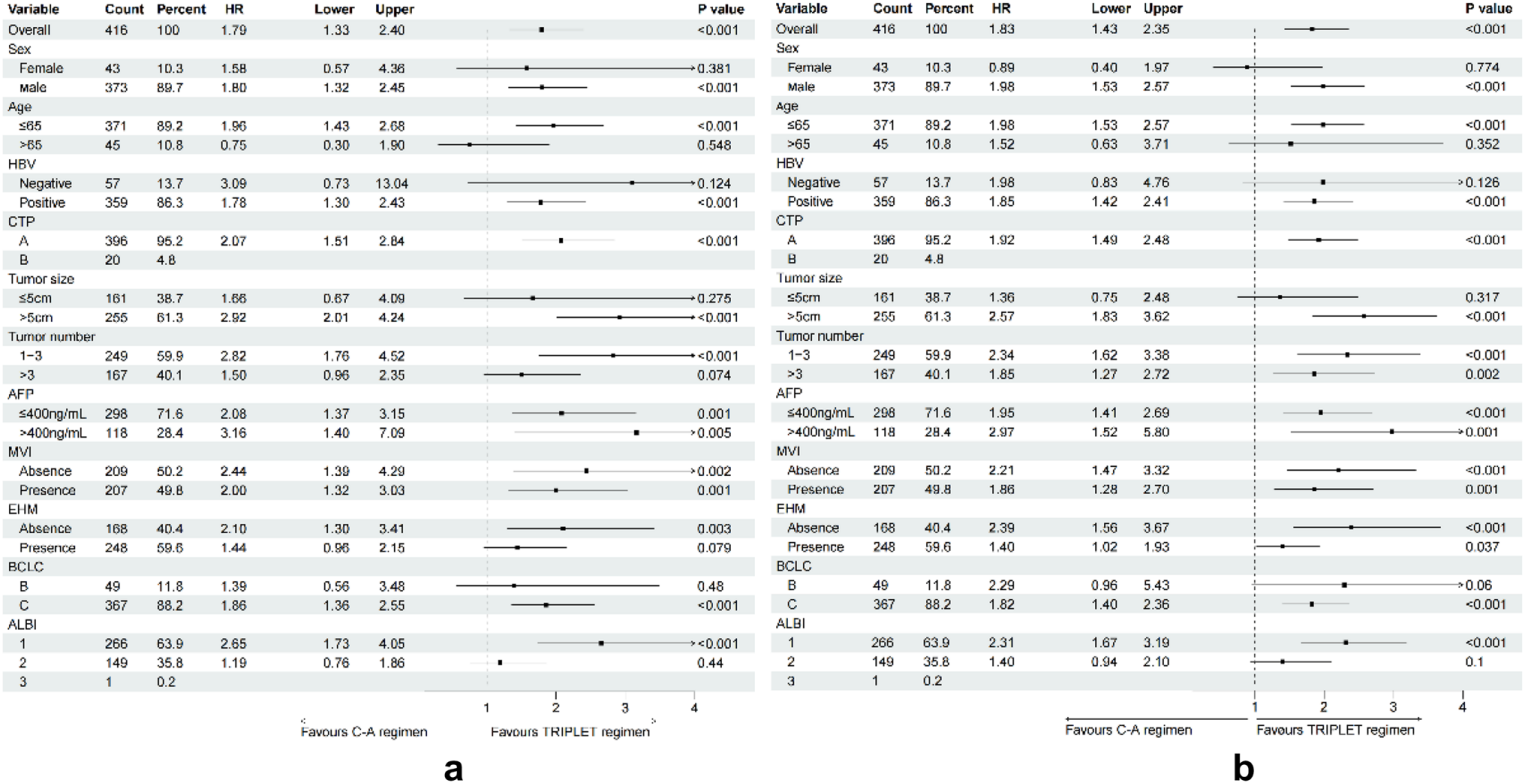
Subgroup analysis of two different two groups. **a** Forest plot for overall survival; **b** Forest plot for progression-free survival. ECOG-PS: Eastern Cooperative Oncology Group; HBV: hepatitis B virus; CTP: Child–Turcotte–Pugh; BCLC: Barcelona Clinic Liver Cancer; HCC: hepatocellular carcinoma; AFP: alpha-fetoprotein

Prior to the PSM, the subgroup analyses of OS and PFS based on clinical variables using forest plots are shown in Fig. 3A and B, which showed that TRIPLET provided a clinical benefit for OS and PFS, outperforming camrelizumab and apatinib in most clinical variables. These results suggested that the TRIPLET appeared to particularly benefit patients with age ≤ 65 years, male, HBV, CTP A, 1–3 tumor number, BCLC C grade (HR: 1.34; 95% CI 1.17–1.54, *p* < 0.001), > 5 cm tumor diameter, without metastasis and ALBI grade 1 based on the OS and PFS. These results provide an interventional treatment strategy for patients with different clinical factors.

### Safety

The adverse event (AE) comparison between the TRIPLET and C–A groups is shown in Table 4. Before PSM, the incidence of patients with AE was 82.3% in the TRIPLET group, which was significantly higher than that in the C–A group (71.3%) (*p* = 0.01). The most common AE was elevated AST in C–A group as well as abdominal pain in the TRIPLET groups. The incidence of patients with grade 3–4 AEs in total was 82.1% in the TRIPLET group, which was higher than that in the C–A group at 71.3% (*p* = 0.01). Moreover, the frequencies of grade 3–4 vomit, abdominal pain, elevated ALT, elevated AST, diarrhea, neutropenia, thrombocytopenia and fatigue were significantly higher in the TRIPLET group than in the C–A monotherapy group. Furthermore, abdominal pain was observed in most of patients in the TRIPLET group after oxaliplatin injection, although the pain was relieved when the injection of oxaliplatin was stopped instantly. Following PSM, there remained no significant difference between the two groups in any grade AE (TRIPLET vs. C–A, 79.8% vs. 60.6%, *p* = 0.08). Meanwhile, the incidence of grade 3–4 AEs in the TRIPLE group was higher than that in the C–A group (78.0% vs. 64.2%, *p* < 0.001). No grade 5 AEs were observed in the study.

**Table 4:** Summary of treatment-related adverse events

Events, *n* (%)	TRIPLET group (*n* = 207)			C–A group (*n* = 209)			Events, *n* (%)	TRIPLET group (*n* = 109)			C–A group (*n* = 109)		
	Before PSM							After PSM					
	Grade 1–2	Grade 3–4	Total	Grade 1–2	Grade 3–4	Total		Grade 1–2	Grade 3–4	Total	Grade 1–2	Grade 3–4	Total
Fever	48 (23.2%)	2 (1.0%)	50 (24.2%)	13 (6.2%)	0 (0%)	13 (6.2%)	Fever	25 (22.9%)	2 (1.8%)	27 (23.4%)	8 (7.3%)	0 (0%)	8 (7.3%)
Nausea	53 (25.6%)	0 (0%)	53 (25.6%)	20 (9.6%)	0 (0%)	20 (9.6%)	Nausea	28 (25.7%)	0 (0%)	28 (25.7%)	13 (11.9%)	0 (0%)	13 (11.9%)
Vomit	31 (15.0%)	8 (3.9%)	39 (18.9%)	7 (3.3%)	1 (0.5%)	8 (3.8%)	Vomit	18 (16.5%)	3 (2.8%)	21 (19.3%)	4 (3.7%)	0 (0%)	5 (4.6%)
Abdominal pain	134 (64.7%)	61 (29.5%)	195 (94.2%)	13 (6.2%)	3 (1.4%)	16(7.6%)	Abdominal pain	70 (64.2%)	27 (24.8%)	97 (89.0%)	6 (5.5%)	1 (0.9%)	7 (6.4%)
Elevated ALT	34 (16.4%)	40 (19.3%)	74 (35.7%)	22 (10.5%)	20 (9.6%)	42 (20.1%)	Elevated ALT	18 (16.5%)	21 (19.3%)	39 (35.8%)	12 (11.0%)	15 (13.8%)	27 (24.8%)
Elevated AST	44 (21.3%)	88 (42.5%)	132 (63.8%)	33 (15.8%)	27 (12.9%)	60 (28.7%)	Elevated AST	23 (21.1%)	48 (44.0%)	71 (65.1%)	20 (18.3%)	13 (11.9%)	33 (30.2%)
Hyperbilirubinemia	13 (6.3%)	14 (6.8%)	27 (13.1%)	14 (6.7%)	15 (7.2%)	29 (13.9%)	Hyperbilirubinemia	8 (7.3%)	6 (5.5%)	14 (12.8%)	6 (5.5%)	7 (6.4%)	13 (11.9%)
Neutropenia	32 (15.5%)	40 (19.3%)	72 (34.8%)	17 (8.1%)	12 (5.7%)	39 (13.8%)	Neutropenia	18 (16.5%)	21 (19.3%)	39 (35.8%)	12 (11.0%)	7 (6.4%)	19 (17.4%)
Thrombocytopenia	51 (24.6%)	48 (23.2%)	99 (47.8%)	28 (13.4%)	21 (10.0%)	49 (23.4)	Thrombocytopenia	23 (21.1%)	30 (27.5%)	53 (28.6%)	16 (14.7%)	10 (9.2%)	26 (23.9%)
Anemia	23 (11.1%)	9 (4.3%)	32 (15.4%)	12 (5.7%)	3 (1.4%)	15 (7.2%)	Anemia	8 (7.3%)	3 (2.8%)	11 (10.1%)	9 (8.3%)	1 (0.9%)	10 (9.2%)
Bleeding	11 (5.3%)	1 (0.5%)	12 (5.8%)	2 (1.0%)	1 (0.5%)	3 (1.5%)	Bleeding	8 (7.3%)	1 (1%)	9 (8.3%)	2 (1.8%)	0 (0%)	2 (1.8%)
Diarrhea	24 (11.6%)	12 (5.8%)	36 (17.4%)	13 (6.2%)	3 (1.4%)	16 (7.6%)	Diarrhea	10 (9.2%)	8 (7.3%)	18 (16.5%)	8 (7.3%)	0 (0%)	8 (7.3%)
Hoarseness	17 (8.2%)	0 (0%)	17 (8.2%)	19 (9.1%)	0 (0%)	19 (9.1%)	Hoarseness	8 (7.3%)	0 (0%)	8 (7.3%)	12 (11.0%)	0 (0%)	12 (11.0%)
Rash	54 (26.1%)	4 (1.9%)	58 (28.0%)	14 (6.7%)	0 (0%)	14 (6.7%)	Rash	28 (25.7%)	3 (2.8%)	31 (28.4%)	7 (6.4%)	0 (0%)	7 (6.4%)
HFS	48 (23.2%)	9 (4.3%)	57 (27.5%)	13 (6.2%)	19 (9.1%)	32 (15.3%)	HFS	31 (28.4%)	3 (2.8%)	34 (31.2%)	8 (7.3%)	8 (7.3%)	16 (14.6%)
Hypertension	56 (27.1%)	27 (13.0%)	83 (40.1%)	35 (16.7%)	21 (10.0%)	56 (26.7%)	Hypertension	28 (25.7%)	15 (13.8%)	43 (39.4%)	23 (21.1%)	36 (33.0%)	59 (54.1%)
RCCEP	47 (22.7%)	0 (0%)	47 (22.7%)	12 (5.7%)	0 (0%)	12 (5.7%)	RCCEP	27 (24.8%)	0 (0%)	27 (24.8%)	7 (6.4%)	0 (0%)	7 (6.4%)
Hypothyroidism	32 (15.5%)	0 (0%)	32 (15.5%)	1 (0.5%)	0 (0%)	1 (0.5%)	Hypothyroidism	16 (14.7%)	0 (0%)	16 (14.7%)	0 (0%)	0 (0%)	0 (0%)
Fatigue	35 (16.9%)	16 (7.7%)	51 (24.6%)	15 (7.2%)	6 (2.9%)	21 (10.1%)	Fatigue	13 (11.9%)	7 (6.4%)	20 (18.3%)	10 (9.2%)	2 (1.8%)	12 (11.0%)
Proteinuria	102 (49.3%)	5 (2.4%)	107 (51.7%)	28 (13.4%)	13 (6.2%)	41 (19.6%)	Proteinuria	56 (51.4%)	2 (1.8%)	58 (53.2%)	15 (13.8%)	6 (5.5%)	21 (19.3%)

PSM: propensity score matching; ALT: alanine aminotransferase; AST: aspartate aminotransferase; HFS: hand–foot syndrome; RCCEP: reactive cutaneous capillary endothelial proliferation

## Discussion

This retrospective cohort study demonstrated that the combined treatment protocol of HAIC plus camrelizumab and apatinib (TRIPLET) is both safe and feasible. In this study, TRIPLET provided a significant OS benefit compared with camrelizumab and apatinib. CARES-301 trial had revealed camrelizumab combined with apatinib was an effective first-line treatment option for uHCC, while the ORR was only 25% and lack of evidence for patients with high tumor burden [15]. Previously, some triple combination treatment protocols have also been registered and are recruiting eligible patients for enrollment. Beyond LEAP-002, the LEAP-012 trial assessed the safety and efficacy of TACE plus lenvatinib plus pembrolizumab in participants with incurable/non-metastatic HCC compared with TACE alone [17]. In addition, the EMERALD-1 global study aimed to evaluate TACE plus durvalumab plus bevacizumab therapy in patients with locoregional HCC compared with TACE plus durvalumab or TACE alone [18]. In this study, we chose HAIC instead of TACE because the standard operating procedure could be standardized and was technically easy to repeat. Furthermore, HAIC avoids most of the uncertain factors affecting TACE, including the lack of standard medication usage, distinctive operating skills, and different operator habits. Therefore, HAIC-oriented combined therapy has the potential to be popularized with a standard dose regimen.

In this study, the ORR, PFS, and OS of TRIPLET treatment outperformed those of current TKI or ICI monotherapy [19], including lenvatinib (ORR in 18.8% and PFS in 7.4 months) in the REFLECT trial [20], nivolumab (ORR in 15% and PFS in 3.7 months) in the CheckMate 459 trial [21], and pembrolizumab (ORR in 18.3% and PFS in 3.0 months) in the KEYNOTE-240 trial [22]. In addition to its superiority to mono-agent therapies, TRIPLET treatment was considered superior to two-agent combined therapies. Compared with the landmark IMbrave-150 trial, which has changed the first-line recommendation of BCLC guidelines, the results from this TRIPLET treatment showed a median PFS of 12.0 months with an ORR of 67.2% as per mRECIST, which is significantly higher than the ORR of 33.2% as per mRECIST and the median PFS of 6.8 months reported by the IMbrave-150 trial [9]. The advantage of TRIPLET is also evident compared with the KEYNOTE-524, LEAP-002 and ORIENT-32 trial, in which lenvatinib and pembrolizumab, as well as sintilimab and bevacizumab-biosimilar were combined for patients with unresectable HCC. Both the estimated median PFS (KEYNOTE-524: 9.7 months, LEAP-002: 7.4 months, ORIENT-32: 4.6 months) and ORR (KEYNOTE-524: 46.3% per mRECIST, LEAP-002: 40.8%, ORIENT-32: 24.3%) of the above trials were lower than those of TRIPLET treatment [10, 12, 23].

In most cases, the patients with high tumor burden have shorter survival and the main cause of death in HCC is intrahepatic tumor progression. The IMbrave-150 study showed that the mOS of patients with high risk (VP4 portal vein invasion, and/or bile duct invasion and/or tumor occupancy of ≥ 50% of liver) is only 7.6 m, which is far shorter than non-high risk group [11]. Combination with HAIC may reduce the tumor burden and prolong survival. In the treatment decision-making of our study, most patients with high tumor burden were recommended local treatment combination with systematic treatment by MDT based on the Guidelines for Diagnosis and Treatment of Primary Liver Cancer in China (2022 Edition), so the tumor burden and AFP level have not been chosen to perform PSM. Even though the TRIPLET group has the lager tumor burden than C–A group, HAIC combined with camrelizumab and apatinib is still associated with better efficacy.

The survival benefit observed in this study may be due to the synergistic antitumor effects of the chemical agents (HAIC of FOLFOX), antiangiogenic agents, and PD-1 inhibitors. Instead of intravenous chemotherapy, HAIC injects chemical reagents directly into the tumor. Oxaliplatin can induce immunogenic cell death by releasing tumor antigens, transporting CRT to the cell surface, and secreting HMGB1 and ATP [24–26]. These molecules related to cell death bind to their respective receptors and support the evolution of tumor-specific CD8^+^ T cells. Indeed, combined antiangiogenic and anti-PD-1/PD-L1 therapy has been shown to elicit T-cell function and drive tumor cells to activate immune checkpoints, thereby generating greater antitumor immunity than anti-PD-1 treatment alone [13]. In addition, the low dose of apatinib therapy (250 mg daily) used in this study has been proven to induce prolonged vascular normalization, thereby reducing tumor hypoxia and acidosis and improving the anti-cancer activity of infiltrating immune cells [27].

During the median follow-up of 25.2 months, we observed increased ORR in the T group compared with those in the C–A group. However, the DCRs were similar between the two groups. In the T group, PD was observed in 71 patients (34.3%), 60.6% (43/71) of whom showed extrahepatic metastasis (EHM). As a result, we considered EHM in patients to indicate a tendency toward PD because HAIC is highly selective for intrahepatic tumors, and some patients with EHM did not achieve no evidence of disease (NED). However, multivariate analysis using the Cox regression model did not identify EHM as an independent prognostic factor for PFS, possibly because of the small sample size of this study. However, for patients who achieved a CR, the duration of the CR has not been reached, indicating satisfactory efficacy prolongation. One patient, who had evaluated as CR after 2 HAIC circles, died from decompensated liver function-related upper gastrointestinal bleeding after three cycles of TRIPLET treatment and subsequently refusing treatment. We also performed sub-analyses for OS and PFS based on common clinical variables. The results indicated that the patients with HCC who received TRIPLET obtained better tumor control and survival benefit than those who received camrelizumab and apatinib in most clinical variables, including ≤ 65 years old, male, ECOG-PS 0, CTP stage A, > 5 cm tumor diameter, and BCLC stage C. Moreover, radiological features, including pseudo-capsulated and infiltrative, are key indicators for combination therapy selection. Wu et al. also provided evidence that HAIC can obtain better outcomes than TACE for infiltrative HCC.

In terms of safety, the T and C–A groups showed some differences in the frequencies and severity of adverse events. The frequencies of both total AEs and grade 3–4 AEs were higher in the T group than those in the C–A group regardless of PSM. Except abdominal pain, the first and second most common grade 3–4 AEs were elevated AST and thrombocytopenia in the T group, which can be explained by chemotherapy-related liver damage and myelosuppression. Moreover, the increased frequencies of abdominal pain, nausea, vomiting, and diarrhea may be caused by chemotherapy, especially for drug diversion to the gastrointestinal tract or cholecyst. Therefore, we sometimes performed gastroduodenal artery embolization during HAIC to reduce drug diversion. Paradoxically, hypertension was the most frequently occurring AE in the C–A group, which was found to a greater extent than that in the T group. One possible explanation for this is that many patients in the T group were treated with chemotherapy, and the side effects, such as fatigue and anemia, counteracted the hypertension caused by the C–A regimen. Even so, we demonstrate that the safety of the TRIPLET scheme in treating advanced HCC was acceptable and similar to the findings of existing studies [16, 28–31].

This study has several limitations that warrant discussion. First, as a retrospective study, the patients were consecutively enrolled, which may have introduced patient selection bias, so some baseline characteristics of patients between two group is different. Second, no biomarker analysis was performed to determine the patients who would benefit most from the TRIPLET combination therapy. Third, the mean follow-up duration in TRIPLET group was not long enough.

## Conclusion

TRIPLET has more promising survival benefits than apatinib and camrelizumab in the management of Ad-HCC, as well as showing tolerable safety. The efficacy and safety of TRIPLET still need to be confirmed by prospective phase III study in the future.

## Supplementary Information

Below is the link to the electronic supplementary material.

Supplementary Material 1.

## Data Availability

The datasets supporting the conclusions of this manuscript are available in the Sun Yat-sen University Cancer Center Research Data Deposit repository (https://www.researchdata.org.cn/).
