# Supplementary material for: Hepatic arterial infusion chemotherapy plus camrelizumab and apatinib for advanced hepatocellular carcinoma
Source: Hepatol Int. 2024 Jul 3;18(5):1486–98. doi: 10.1007/s12072-024-10690-6 (PMC11461759; doi:10.1007/s12072-024-10690-6)
Supplement: Supplementary file 1 — Supplementary Material 1. [file 12072_2024_10690_MOESM1_ESM.docx]

**Hepatic arterial infusion chemotherapy plus Camrelizumab and Apatinib for Advanced Hepatocellular Carcinoma**

**This supplementary material includes:**

**1.Supplementary Methods**

**E1.1 Criteria for protocol treatment discontinuation**

1. Tumor progression. The progression disease (PD) were assessed by enhanced dynamic CT or MRI based on modified Response Evaluation Criteria in Solid Tumor (mRECIST).
2. Intolerable adverse event

i) Patient cannot resume IAT, TKI, or ICI after 30 days of interruption due to an adverse event;

ii) An adverse event that meets the criteria for dose reduction occurs after the dose was already reduced to the lowest level;

iii) Life-threatening adverse event;

C)The need for another anticancer treatment due to downstaging at the physician’ s discretion;

D) IAT becomes technically infeasible;

E) Inadequate blood or bone marrow (leukopenia count < 3.0×10^9^/L, platelet count < 50×10^9^/L, and hemoglobin < 8.0 g/L)

1. Patient requests to discontinue the study；
2. Death.

**E1.2 Definitions of Variables**

In this study, 38 clinical variables are collected as follows: (1) demographic and history variables (ECOG, pathology differentiation, weight，height, BMI, age, gender, comorbidities (i.e., hypertension, diabetes, heart disease, renal disease a esophageal gastric varices, etc.), etiology, CTP class, ALBI grade, ascites); (2) tumor features (maximal tumor diameter, number of tumor, tumor burden, macroscopic vascular invasion, metastasis, and BCLC stage); (3) laboratory findings (α-fetoprotein [AFP], des-γ-carboxy prothrombin [DCP], serum albumin; [ALB], total bilirubin [TB], platelet counts, prothrombin time (PT), international normalized ratio [INR], aspartate aminotransferase [AST] and alanine aminotransferase [ALT]), C reactive protein (CRP), creatinine, neutrophils, lymphocyte. Albumin- bilirubin (ALBI) grades were used to replace CTP grade for their objectiveness. ALBI score was calculated before treatment using the appropriate clinical parameters and ALBI grade was defined as follows: (log 10 bilirubin [BI] [μ mol /L] × 0.66) + (albumin [AL] [g/L] ×-0.085), (grade 1, 2, and 3 = ≤ -2.60, > -2.60 to -1.39, and > -1.39, respectively). For more detailed evaluations of patients with the middle grade of ALBI (grade 2), we used modified ALBI (mALBI) grading consisting of 4 levels, which included subgrading for the middle grade of 2 (2a and 2b) based on an ALBI score of –2.27 as the cut-off, which was previously reported as the value for indocyanine green retention after 15 min (ICG-R15) of 30%^[4]^ ; (4) treatment parameters (IAT modalities, combination with TKI, combination with ICI, sequential local therapy and the response of first IAT). The responses to IAT was assessed by dynamic contrast enhanced CT or magnetic resonance imaging (MRI) based on modified Response Evaluation Criteria in Solid Tumor (mRECIST), including complete response (CR), partial response (PR), stable disease (SD), and progression disease (PD), which was performed every 4–6 weeks after initial IAT and evaluated independently by two radiologists (reader 1, L.Z.L., and reader 2, J. Z., with 10 years of experience) who were blinded to IAT procedures at the time of data collection.

**2.Supplementary Tables**

**Table S1.** Post-study treatment in follow-up.

|  | **T group**  **(n=207)** | **C-A group**  **(n=209)** | **P-Value** |
| --- | --- | --- | --- |
| Number of patients with at least one treatment after disease downstaging, for maintaining the response of study treatment, or due to the intolerable toxicity |  |  |  |
| Absence | 941 (67.7) | 1071 (72.8) |  |
| Presence | 448 (32.3) | 400 (27.2) |  |
| Cancer disappeared | 38 (2.8) | 40(2.7) |  |
| Thermal ablation | 56(4.1) | 187(12.6) |  |
| Surgical resection | 124(9.0) | 32(2.2) |  |
| Radiotherapy for vascular invasion | 45(3.3) | 24(1.6) |  |
| TACE or HAIC | 47(3.4) | 26(1.7) |  |
| TKIs | 486(35.2) | 331(22.3) |  |
| ICIs | 369(26.8) | 151(10.2) |  |
| TKIs plus ICIs | 214(15.5) | 128(8.6) |  |
| Number of patients with at least one 2-line treatment after disease progression | 191 (13.9) | 378 (25.5) |  |
| Thermal ablation | 9(0.7) | 12(0.8) |  |
| Surgical resection | 11(0.8) | 8(0.5) |  |
| Radiotherapy for vascular invasion | 8(0.6) | 6(0.4) |  |
| TACE or HAIC | 12(0.9) | 16(1.0) |  |
| TKI | 25(1.8) | 36(2.4) |  |
| ICI | 15(1.1) | 14(0.9) |  |
| Number of patients with at least one 3-line treatment after disease progression | | | |
|  | 112(8.1) | 285(19.2) |  |
| Thermal ablation | 6(0.4) | 5 (0.3) |  |
| Surgical resection | 0(0) | 0(0) |  |
| Radiotherapy for vascular invasion | 2(0.1) | 3(0.2) |  |
| TACE | 5(0.4) | 3(0.2) |  |
| TKIs | 17(1.2) | 11(0.7) |  |
| ICIs | 5(0.4) | 8(0.5) |  |
| TKIs plus ICIs | 4(0.3) | 2(0.1) |  |
| TACE, transarterial chemoembolization; HAIC, hepatic arterial infusion chemotherapy; ICIs, immune checkpoint inhibitors; TKIs, tyrosine kinase inhibitors; MWA, microwave ablation; RFA, radiofrequency ablation; SBRT, stereotactic body radiation therapy | | | |

**Reference**

1. Ueshima K, Komemushi A, Aramaki T, Iwamoto H, Obi S, Sato Y, Tanaka T, Matsueda K, Moriguchi M, Saito H, Sone M, Yamagami T, Inaba Y, Kudo M, Arai Y. Clinical Practice Guidelines for Hepatic Arterial Infusion Chemotherapy with a Port System Proposed by the Japanese Society of Interventional Radiology and Japanese Society of Implantable Port Assisted Treatment. Liver Cancer. 2022 May 5;11(5):407-425. doi: 10.1159/000524893.

2. Yamasaki T, Saeki I, Yamauchi Y, Matsumoto T, Suehiro Y, Kawaoka T, Uchikawa S, Hiramatsu A, Aikata H, Kobayashi K, Kondo T, Ogasawara S, Chiba T, Takami T, Chayama K, Kato N, Sakaida I. Management of Systemic Therapies and Hepatic Arterial Infusion Chemotherapy in Patients with Advanced Hepatocellular Carcinoma Based on Sarcopenia Assessment. Liver Cancer. 2022 Feb 22;11(4):329-340. doi: 10.1159/000522389.

3. Wang T, Dong J, Zhang Y, Ren Z, Liu Y, Yang X, Sun D, Wang Y. Efficacy and safety of hepatic artery infusion chemotherapy with mFOLFOX in primary liver cancer patients with hyperbilirubinemia and ineffective drainage: a retrospective cohort study. Ann Transl Med. 2022 Apr;10(7):411. doi: 10.21037/atm-22-978.

4.Hiraoka A, Kumada T, Tsuji K, Takaguchi K, Itobayashi E, Kariyama K, et al. Validation of Modified ALBI Grade for More Detailed Assessment of Hepatic Function in Hepatocellular Carcinoma Patients: A Multicenter

Analysis. Liver Cancer. 2019 Mar;8(2):121–129.
